# MIR4435-2HG Is a Potential Pan-Cancer Biomarker for Diagnosis and Prognosis

**DOI:** 10.3389/fimmu.2022.855078

**Published:** 2022-06-15

**Authors:** Chenming Zhong, Zijun Xie, Ling-hui Zeng, Chunhui Yuan, Shiwei Duan

**Affiliations:** ^1^ Department of Clinical Medicine, School of Medicine, Zhejiang University City College, Hangzhou, China; ^2^ Medical Genetics Center, School of Medicine, Ningbo University, Ningbo, China; ^3^ Institute of Translational Medicine, Zhejiang University City College, Hangzhou, China

**Keywords:** MIR4435-2HG, cancer, competing endogenous RNA, diagnosis, prognosis

## Abstract

The lncRNA MIR4435-2 host gene (MIR4435-2HG) is located on human chromosome 2q13, and its expression is up-regulated in 18 tumors. MIR4435-2HG participates in 6 signaling pathways to promote tumorigenesis, including the TGF-β signaling pathway, Wnt/β-catenin signaling pathway, MDM2/p53 signaling pathway, PI3K/AKT signaling pathway, Hippo signaling pathway, and MAPK/ERK signaling pathway. MIR4435-2HG competitively binds with 20 miRNAs to form a complex ceRNA network, thereby regulating the expression of downstream target genes. The high expression of MIR4435-2HG is also closely related to the clinicopathological characteristics and poor prognosis of a variety of tumors. Also, the high expression of MIR4435-2HG in peripheral blood or serum has the value of predicting the risk of 9 tumors. In addition, MIR4435-2HG participates in the mechanism of action of three cancer drugs, including resveratrol for the treatment of lung cancer, cisplatin for non-small cell lung cancer and colon cancer, and carboplatin for triple-negative breast cancer. This article systematically summarizes the diagnostic and prognostic value of MIR4435-2HG in a variety of tumors and outlines the ceRNA network and signaling pathways related to MIR4435-2HG, which will provide potential directions for future MIR4435-2HG research.

## Introduction

Long non-coding RNAs (lncRNAs) are transcripts longer than 200 nucleotides that can not be translated into proteins. With the rapid development of high-throughput sequencing technology, more and more lncRNAs have been reported to participate in tumor differentiation, stemness, migration, invasion, apoptosis, and proliferation (1).

The lncRNA MIR4435-2 host gene (MIR4435-2HG) is located on human chromosome 2q13, also known as lncRNA-AWPPH, LINC00978, and AK001796. In 2015, MIR4435-2HG was first discovered to be involved in the cell growth inhibition of resveratrol in lung cancer (2). At present, MIR4435-2HG has been proven to be an oncogenic lncRNA, and its abnormal up-regulation can promote the occurrence and development of 18 tumors. In addition, MIR4435-2HG is abnormally up-regulated in the blood of patients with at least 9 tumors, suggesting that MIR4435-2HG can be used as a non-invasive diagnostic marker for these 9 tumors.

Competitive endogenous RNA (ceRNA) can sponge miRNA to regulate downstream mRNA of miRNA (3). MIR4435-2HG is the ceRNA of 20 miRNAs, which can regulate many downstream genes. MIR4435-2HG participates in at least 6 signaling pathways, including TGF-β signaling pathway, Wnt/β-catenin signaling pathway, MDM2/p53 signaling pathway, PI3K/AKT signaling pathway, Hippo signaling pathway, and MAPK/ERK signaling pathway.

The abnormal up-regulation of MIR4435-2HG is closely related to the clinicopathological characteristics of 11 tumors, including tumor size, TNM stage, lymph node metastasis, etc. The high expression of MIR4435-2HG12 is associated with the poor prognosis of 12 tumors. In addition, MIR4435-2HG is related to the mechanism of action of cancer drugs, including resveratrol for the treatment of lung cancer (2), cisplatin for non-small cell lung cancer and colon cancer (4, 5), and carboplatin for triple-negative breast cancer (6).

There is no comprehensive overview related to MIR4435-2HG. Here, this article summarizes the diagnostic and prognostic value of MIR4435-2HG in tumors, clarifies its gene regulatory network, and discusses the future directions and challenges of MIR4435-2HG research.

## Abnormal Regulation and Biological Effects of MIR4435-2HG in Cancers

### Pan-Cancer Analysis of MIR4435-2HG Using TCGA Database

We downloaded the expression data of MIR4435-2HG in TCGA, TARGET, and GTEx of 32 cancer types from the UCSC Xena (https://xenabrowser.net/) database, and further performed log2(x+1) transform for the expression data.

**Figure 1:**
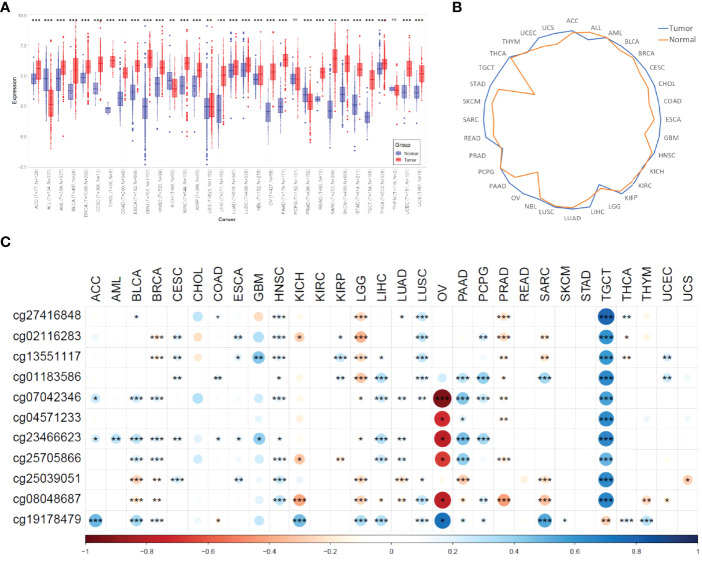
A pan-cancer analysis of MIR4435-2HG. **(A)** MIR4435-2HG is dysregulated in 32 cancer types. (*** means p < 0.001, ** means p<0.01, * means p<0.05, ns means no significant difference); **(B)** quantile expression of MIR4435-2HG in 32 cancer types; **(C)** The correlation tests between MIR4435-2HG expression and methylation of MIR4435-2HG CpG sites (*** means p<0.001, ** means p<0.01, * means p<0.05). ACC, Adrenocortical carcinoma; ALL, Acute lymphoblastic leukemia; AML, Acute myeloid leukemia; BLCA, Bladder urothelial carcinoma; BRCA, Breast invasive carcinoma; CESC, Cervical squamous cell carcinoma and endocervical adenocarcinoma; CHOL, Cholangiocarcinoma; COAD, Colon adenocarcinoma; ESCA, Esophageal carcinoma; GBM, Glioblastoma multiforme; HNSC, Head and Neck squamous cell carcinoma; KICH, Kidney chromophobe; KIRC, Kidney renal clear cell carcinoma; KIRP, Kidney renal papillary cell carcinoma; LGG, Brain lower grade glioma; LIHC, Liver hepatocellular carcinoma; LUAD, Lung adenocarcinoma; LUSC, Lung squamous cell carcinoma; NBL, Neuroblastoma; OV, Ovarian serous cystadenocarcinoma; PAAD, Pancreatic adenocarcinoma; PCPG, Pheochromocytoma and Paraganglioma; PRAD, Prostate adenocarcinoma; READ, Rectum adenocarcinoma; SARC, Sarcoma; STAD, Stomach adenocarcinoma; SKCM, Skin cutaneous melanoma; TGCT, Testicular germ cell tumors; THCA, Thyroid carcinoma; THYM, Thymoma; UCEC, Uterine corpus endometrial carcinoma; UCS, Uterine carcinosarcoma.

We compared the expression differences of MIR4435-2HG between normal and tumor samples in each cancer type using the unpaired Wilcoxon Test method of R software (version 4.1.1). As shown in **Figure 1A**, we observed significant upregulation of MIR4435-2HG in 27 tumors, significant downregulation of MIR4435-2HG in 3 tumors (ALL, PRAD, and KICH), and no significant difference in 2 tumors (PCPG and THYM). In addition, we evaluated the median expression of MIR4435-2HG among all ncRNAs in 32 tumors and corresponding non-tumor tissues. As shown in **Figure 1B**, expression of MIR4435-2HG exceeded at least 75% of lncRNAs in all tumors, suggesting the value of MIR4435-2HG in pan-cancer.

### Research Progress of MIR4435-2HG Related Studies

At present, MIR4435-2HG has been confirmed to be an oncogenic lncRNA of 18 cancers. These cancers involve the digestive, respiratory, reproductive, urinary, and nervous systems in humans (**Table 1**). Among them, the experimental results in most tumors were consistent with the bioinformatic analysis results, except for ALL and prostate cancer.

**Table 1:** Expression level and biological functions of MIR4435-2HG in human cancers.

System	Tumor type	Sample size	Assessed cell lines	Animals	Expression	Related genes	Effect *in vitro*	Effect *in vivo*	Ref.
Digestive system	OSCC	Blood specimens of 44 OSCC patients and 38 healthy controls	OSCC (SCC25 (HPV negative) and SCC090 (HPV positive))		Upregulation	TGF-β1↑ E-cadherin↓	proliferation↑ migration↑ invasion↑		(7)
	ESCC	175 pairs of tissues			Upregulation				(8)
		50 pairs of tissues	ESCC (Eca-109 and TE-1)	6 BALB/c nude mice (5-week-old)	Upregulation	MDM2↑ p53↓ p21↓	proliferation↑ cell cycle↑ apoptosis↓		(9)
	GC	57 pairs of tissues	GC (SNU5, HGC-27, and SGC-7901); Normal (GES-1)	10 athymic BALB/c mice (3 to 4-week-old, male)	Upregulation	N-cadherin↑ Vimentin↑ MMP9↑ VEGF↑ α-SMA↑ MYC↑ β-catenin↑ CCND1↑ DSP↓ E-cadherin↓	proliferation↑ migration↑ invasion↑ cell cycle↑ EMT↑ apoptosis↓	tumor growth↑	(10)
		150 pairs of tissues	GC (BGC-823, AGS, SGC-7901, and MGC-803); Normal (GES-1)		Upregulation	miR-497-5p↓/NTRK3↑; CDK6↑ CDK4↑ CCND1↑ MYC↑ N-cadherin↑ Vimentin↑ MMP-9↑ MMP-3↑ MMP-2↑ Bcl-2↑ Mcl-1↑ E-cadherin↓ Bim↓ Bax↓	proliferation↑ migration↑ invasion↑ apoptosis↓		(11)
		72 pairs of tissues; Serum specimens of 50 GC patients and 50 healthy controls	GC (MGC-803, SGC-7901, BGC-823, and HGC-27); Normal (GES-1)	10 BALB/c nude mice (4-week-old, male)	Upregulation	TGF-β1↑ SMAD2↑ MMP9↑ Bcl-2↑ p21↓	proliferation↑ migration↑ invasion↑ cell cycle↑ EMT↑ apoptosis↓	tumor growth↑	(12)
		343 GC tissues and 30 normal tissues (from TCGA database); 60 pairs of tissues (from 18 patients)	GC (HGC-27, AGS, SGC-7901, and MGC-803); Normal (GES-1)	10 nude athymic mice (5 to 6-week-old, male)	Upregulation	miR-138-5p↓/Sox4↑; Vimentin fibronectin↑ Sox4↑ E-cadherin↓	proliferation↑ migration↑ invasion↑ EMT↑	tumor growth↑	(53)
		40 pairs of tissues	GC (HGC-27, BGC-823, SGC-7901, and MKN-45); Normal (GES-1)		Downregulation	miR-203a-3p↑/DKK2↓	proliferation↓ invasion↓		(13)
	CRC	90 pairs of tissues	CRC (HT29, SW620, LoVo, LS123, and HCT116); Normal (NCM460)	10 nude athymic BALB/c (nu/nu) mice (5-week-old, male)	Upregulation	miR-206-3p↓/YAP1↑; CTGF↑ AREG↑ Vimentin↑ Snail↑ Slug↑ Twist1↑ E-cadherin↓	proliferation↑ migration↑ invasion↑ EMT↑	tumor growth↑	(1)
		102 pairs of tissues	CRC (LoVo, SW620, SW480, LS174T, HCT116, and HT29); Normal (HUVEC)		Upregulation		proliferation↑ apoptosis↓		(14)
		70 pairs of tissues			Upregulation	β-catenin↑			(15)
	Colon cancer	Serum specimens of 46 colon cancer patients and 42 healthy controls			Upregulation	GLUT-1↑	proliferation↑		(16)
	COAD	86 pairs of tissues; Whole blood specimens of 86 patients and 56 healthy controls	Colorectal adenocarcinoma (HT-29, Hs 698.T, and SNU-C1)		Upregulation	TGF-β1↑	proliferation↑		(17)
	HCC	22 pairs of tissues	HCC (Huh7, SMMC7721, BEL-7402, and HepG2); Normal (LO2)	6 nude mice	Upregulation	miR-22-3p↓/YWHAZ↑	proliferation↑ migration↑ invasion↑	tumor growth↑	(18)
		64 pairs of tissues	HCC (SNU-398 and SNU-182)		Upregulation	miR-487a↑	proliferation↑		(19)
		49 pairs of tissues	HCC (SK-Hep1, Bel-7404, Huh7, Hep3B, and HepG2); Normal (LO2)	24 BALB/c nude mice (male)	Upregulation	ERK↑ p38↑ JNK↑	proliferation↑ cell cycle↑ apoptosis↓	tumor growth↑	(20)
		120 pairs of tissues (from GEO database); 33 pairs of tissues (from 33 patients); Serum specimens of 58 HCC patients, 49 liver benign disease patients and 45 healthy controls	HCC (7721, 7402, HepG2, and LM3); Normal (7702)	10 BALB/c nude mice (4-week-old, male)	Upregulation	EZH2↑ Bcl-2↑ CCND1↑ N-cadherin↑ Vimentin↑ Slug↑ Snail↑ Twist1↑ p21↓ E-cadherin↓	proliferation↑ migration↑ invasion↑ cell cycle↑ EMT↑ apoptosis↓	tumor growth↑ metastasis↑	(21)
		88 pairs of HCC tissues and 20 PVTT tissues	HCC (SMMC-7721, HCCLM3, Huh7, and HepG2); Normal (QSG-7701)	12 athymic BALB/c nude mice (male)	Upregulation	Snail1↑ PI3K↑	proliferation↑ migration↑	tumor growth↑ metastasis↑	(22)
		73 pairs of tissues	HCC (SMMC-7721, Huh-7, MHCC-97H, and MHCC-97L);		Upregulation		proliferation↑ invasion↑		(23)
			Normal (LO2)						
Respiratory system	LC	52 pairs of tissues	Lung cancer (A549, H1770, H596, H1975, H1650, and H1299); Normal (16HBE)	2 athymic BALB/c nude mice (4 to 6-week-old, female)	Upregulation	N-cadherin↑ Vimentin↑ N-Twist1↑ E-cadherin↓	proliferation↑ migration↑ invasion↑ EMT↑ cancer stem cell traits↑	tumor growth↑ metastasis↑	(24)
		42 pairs of tissues	Lung cancer (A549 and H446); Normal (BEAS2B and 16HBE)	16 BALB/c nude mice (5-week-old)	Upregulation		proliferation↑ cell cycle↑	tumor growth↑	(2)
	NSCLC	39 pairs of tissues	NSCLC (H1299, H1650, A549, and PC9); Normal (16HBE)		Upregulation	miR-6754-5p↓	proliferation↑ migration↑ invasion↑ apoptosis↓		(25)
		Number not shown	Lung cancer (A549, NCI-H1650, and HCC827); Normal (HBE)		Upregulation	miR-204-5p↓/CDK6↑	proliferation↑ migration↑ invasion↑		(26)
		Lung tissues and serum specimens of 138 NSCLC patients and 32 healthy controls	NSCLC (H1581 and H1993); Normal (NuLi-1)		Upregulation	TGF-β1↑	migration↑ invasion↑		(27)
		Blood specimens of 128 NSCLC patients and 30 healthy controls	NSCLC (H1993 and H2170); Normal (IMR-90)		Upregulation	TGF-β1↑	migration↑ invasion↑		(28)
		88 pairs of tissues; Serum specimens of 88 NSCLC patients and 88 healthy controls	NSCLC (NCI-H23 and NCI-H522); Normal (WI-38)		Upregulation	β-catenin↑	proliferation↑ apoptosis↓		(29)
		Blood specimens of 26 SCLC patients, 29 patients NSCLC patients, and 32 healthy controls	Lung cancer (H1770, A549, H1975, H596, H1299, and H1650); Normal (BEAS2B)		Upregulation	TGF-β1↑	proliferation↑ migration↑		(30)
Reproductive system	OC	58 pairs of tissues; Serum specimens of 58 OC patients and 46 healthy controls	OC (UWB1.289 and UWB1.289+BRCA1)		Upregulation	β-catenin↑	proliferation↑ migration↑ invasion↑		(31)
		23 pairs of tissues; Plasma specimens of 66 OC patients and 54 healthy controls	OC (UWB1.289 and UWB1.289+BRCA1)		Upregulation	TGF-β1↑	migration↑ invasion↑		(32)
		42 pairs of tissues	OC (SKOV3, Caov-3, A2780, and OVCAR3); Normal (ISOE80)	6 BALB/c nude mice (4-week-old, female)	Upregulation	miR-128-3p↓/CKD14↑; Bcl-2↑ Vimentin↑ E-cadherin↓	proliferation↑ migration↑ invasion↑ EMT↑ apoptosis↓	tumor growth↑	(33)
	CC	306 CESC and 13 normal tissues (from TCGA database); 59 pairs of tissues (from 59 patients)	CC (siha/hela); Normal (HCerEpiC)		Upregulation	miR-128-3p↓/MSI2↑	proliferation↑ migration↑ invasion↑		(34)
	BC	1085 breast cancer tissues and 112 normal tissues (from GEPIA database)	Breast cancer (MDA-MB-231 MCF-7); Normal (MCF-10A)		Upregulation	β-catenin↑ N-cadherin↑ Vimentin↑ ZEB1↑ Bcl2↑ PCNA↑ E-cadherin↓ Bax↓	proliferation↑ migration↑ invasion↑ apoptosis↓		(35)
		195 breast cancer patients; 36 pairs of tissues	Breast cancer (T47D, ZR751, MCF-7, MDA-MB-453, BCAP37, ZR7530, MDA-MB-436, SKBR3, MDA-MB-468, MDA-MB-231, MDA-MB-231HM, and BT549); Normal (MCF10A)		Upregulation				(36)
	TNBC	68 pairs of tissues; Plasma specimens of 68 TNBC patients and 62 healthy controls	TNBC (MDA-MB-231 and BT-20)		Upregulation	FZD7↑	proliferation↑		(37)
		Plasma specimens of 72 TNBC patients 44 healthy controls	TNBC (MDA-MB-231 and BT-20)		Upregulation		proliferation↑		(6)
	PCa	Blood specimens of 68 PCa patients and 62 healthy controls	PCa (22Rv1)		Upregulation	TGF-β1↑	migration↑ invasion↑		(38)
			PCa (VCaP, LNCaP, DU145, and PC-3); Normal (WPMY-1)	20 nude mice were (6 to 8-week-old, male)	Upregulation	ST8SIA1↑ β-catenin↑ MYC↑ CCND1↑	proliferation↑ migration↑ invasion↑	tumor growth↑	(39)
Urinary system	ccRCC	40 pairs of tissues	ccRCC (786-O, 769-P, Caki-1, Caki-2, ACHN, and A498); Normal (HK-2)	3 BALB/c (nu/nu) nude mice (4 to 6-week-old, female)	Upregulation	miR-513a-5p↓/KLF6↑	proliferation↑ invasion↑	tumor growth↑	(3)
		118 pairs of tissues	ccRCC (786-O and OSRC-2); Normal (HK-2)	16 athymic nude mice (6-week-old, male)	Upregulation	PC↑	proliferation↑ migration↑ invasion↑ cell cycle↑ EMT↑ apoptosis↓		(40)
	BCa	60 pairs of tissues	BCa (T24, J82, UMUC3, and 5637); Normal (SV-HUC-1)		Upregulation	miR-4288-3p↓	proliferation↑ migration↑ invasion↑		(41)
Nervous system	Glioma	Plasma specimens of 34 metastatic glioma patients, 32 non-metastatic glioma patients, and 42 healthy controls	Glioma (Hs 683 and CCD-25Lu)		Upregulation	HIF1α↑	migration↑ invasion↑		(42)
		48 glioma tissues; Plasma specimens of 22 metastatic glioma patients, 26 non-metastatic glioma patients, and 38 healthy controls	Glioma (Hs 683 and CCD-25Lu)		Upregulation	TGF-β1↑	migration↑ invasion↑		(43)
	GBM	163 GBM tissues and 207 normal tissues (from GEPIA database); 40 pairs of tissues (from 40 patients)	GBM (LN229, U87MG, U87, and U251); Normal human astrocytes (NHAs)	10 athymic BALB/c mice (4 to 5-week-old, male)	Upregulation	miR-1224-5p↓/TGFBR2↑	proliferation↑ invasion↑	tumor growth↑	(44)
Others	Osteosarcoma	36 pairs of tissues	Osteosarcoma (U2OS, SAOS2, HOS, and 143B); Normal (hFOB 1.19)		Upregulation	miR-93-3p↓/FZD7↑,	proliferation↑ migration↑ invasion↑		(45)
						MYC↑ SOX4↑ CCND1↑			
		30 pairs of tissues	Osteosarcoma (MG-63 and U2OS); Normal (hFOB1.19)		Upregulation	Bcl-2↑ Bax↓	proliferation↑ migration↑ invasion↑ apoptosis↓		(46)
	HNSC	519 HNSCC tissues and 44 normal tissues (from GEPIA database); 18 pairs of tissues (from 18 patients)	HNSCC (CAL27 and SCC25)	10 BALB/c nude mice (4-week-old)	Upregulation	miR-383-5p↓/RBM3↑,	proliferation↑ migration↑ invasion↑ EMT↑	tumor growth↑	(47)
						Vimentin ↑ E-cadherin↓			
	Melanoma	28 pairs of tissues	Melanoma (A375 and A2058); Normal (HEMa-LP)		Upregulation	miR-802-5p↓/FLOT2↑	proliferation↑ migration↑ invasion↑		(48)
	T-ALL	Bone marrow with malignant cells of 32 pediatric T-ALL patients and 32 healthy controls	T-ALL (Loucy)		Upregulation	ROCK2↑	proliferation↑ apoptosis↓		(49)
	NPC		NPC (5-8F, CNE1, CNE2, and HONE1); Normal (HNEpC)		Upregulation	LSD1 (in cytoplasm)↑ PTEN (in nucleus)↓	proliferation↑ migration↑ apoptosis↓		(50)

OSCC, oral squamous cell carcinoma; ESCC, esophageal squamous-cell carcinoma; GC, gastric cancer; CRC, colorectal cancer; COAD, Colon adenocarcinoma; HCC, hepatocellular carcinoma; PVTT, portal vein tumor thrombus; LC, lung cancer; SCLC small cell lung cancer; NSCLC, nonsmall cell lung cancer; OC, ovarian cancer; CC, cervical cancer; BC, breast cancer; TNBC, triple-negative breast cancer; PCa, prostate carcinoma; ccRCC, clear cell renal cell carcinoma; BCa, bladder cancer; GBM, glioblastoma; HNSC, head and neck squamous cell carcinoma; T−ALL, T−cell acute lymphoblastic leukemia; NPC, nasopharyngeal carcinoma.

↑, Promotion; ↓, Inhibition.

**Figure 2:**
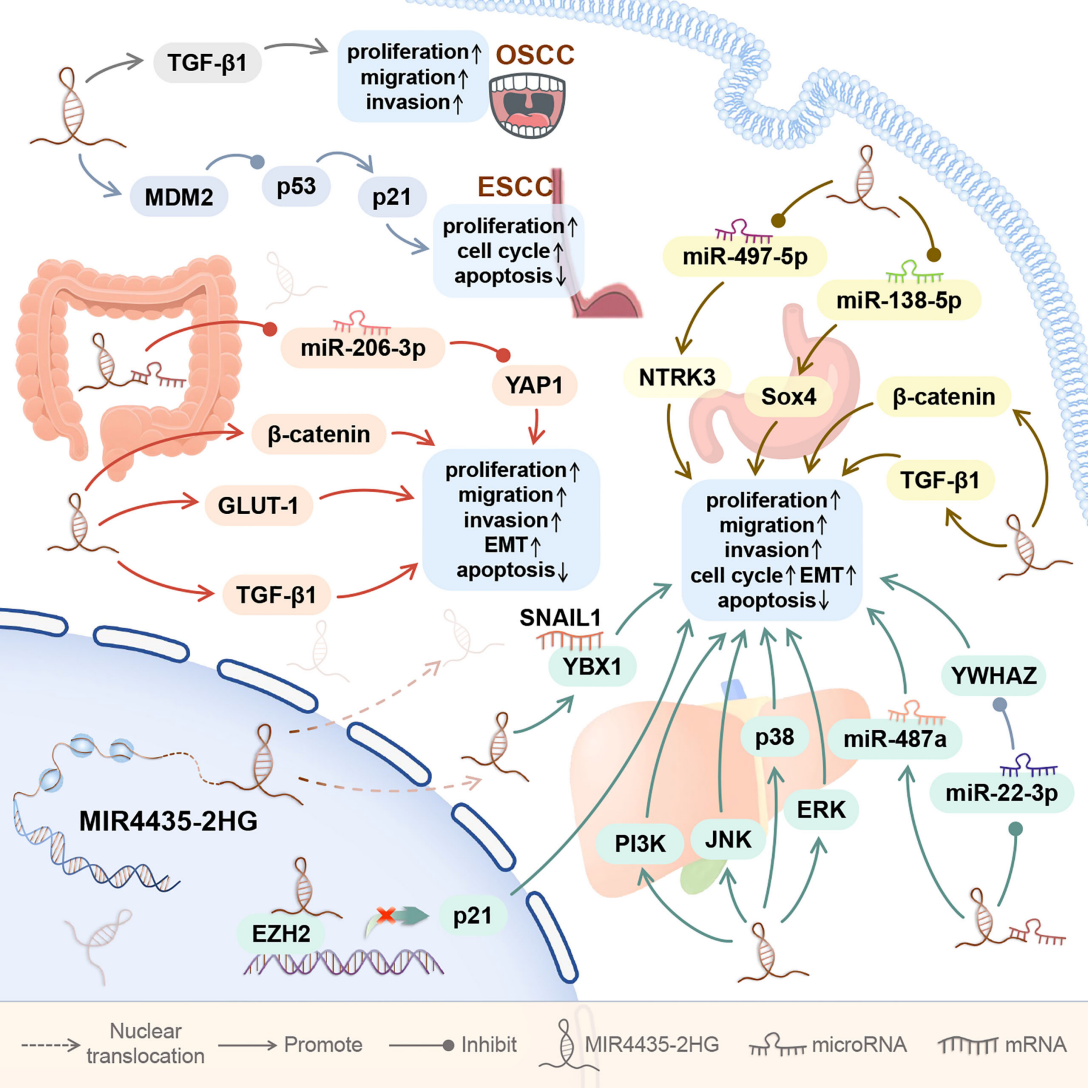
The role of MIR4435-2HG in digestive system cancer. In the digestive system, MIR4435-2HG can promote the growth of 5 types of tumors, including oral squamous cell carcinoma (OSCC), esophageal squamous cell carcinoma (ESCC), hepatocellular carcinoma (HCC), gastric cancer (GC), and colorectal cancer (CRC). By regulating downstream genes, MIR4435-2HG can affect tumor cell proliferation, migration, invasion, apoptosis, EMT, and cell cycle.

As shown in **Figure 2**, the expression of MIR4435-2HG is up-regulated in the five digestive system cancers. MIR4435-2HG is highly expressed in blood and tumor cell lines of oral squamous cell carcinoma (OSCC) (7), in tumor tissues and tumor cell lines of esophageal squamous cell carcinoma and hepatocellular carcinoma (8, 9, 18–23), and in serum, tumor tissues, and tumor cell lines of gastric cancer and colorectal cancer (1, 10–12, 14–17).

MIR4435-2HG is highly expressed in lung cancer tissues and tumor cell lines (2, 24–27, 29). Abnormal up-regulation of MIR4435-2HG was also detected in whole blood and serum of non-small cell lung cancer patients (27–30).

Among reproductive system cancers, MIR4435-2HG is highly expressed in tumor tissues and tumor cell lines of ovarian cancer (31–33), cervical cancer (34), and breast cancer (6, 35–37). In addition, MIR4435-2HG is highly expressed in prostate cancer cell lines and whole blood (38, 39), in serum and plasma of ovarian cancer patients (31, 32), and in plasma of triple negative breast cancer (TNBC) patients (6, 37).

**Figure 3:**
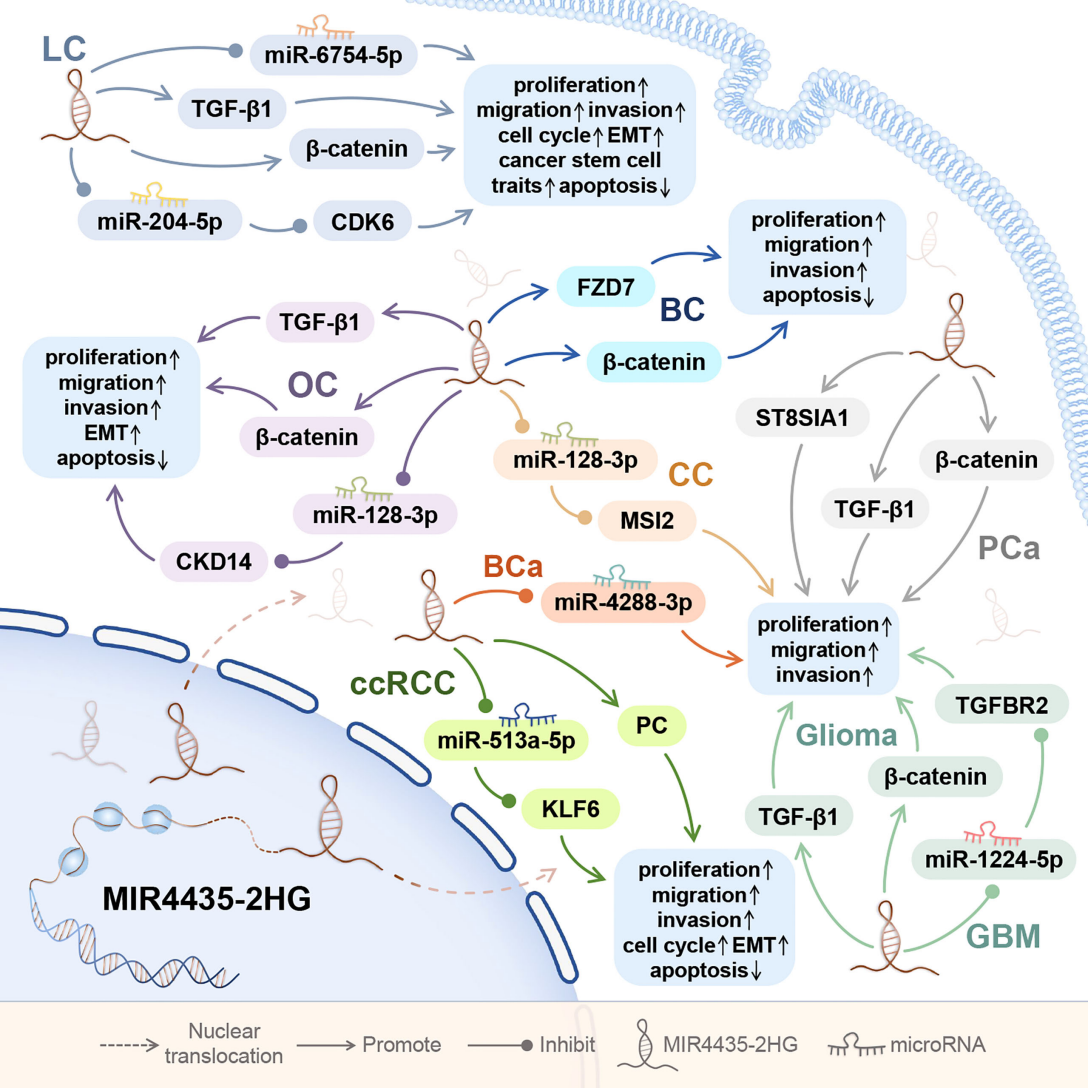
The role of MIR4435-2HG in tumors of the respiratory system, reproductive system, urinary system, and nervous system. MIR4435-2HG can also affect the proliferation, migration, invasion, apoptosis, EMT, and cell cycle of tumor cells by regulating downstream genes. Tumor of the respiratory system consists of lung cancer (LC); Tumors of the reproductive system consist of ovarian cancer (OC), cervical cancer (CC), breast (BC), and prostate cancer (PCa); Tumors of the urinary system include clear cell renal cell carcinoma (ccRCC) and bladder cancer (BCa); and tumor of the nervous system includes gliomas.

In tumors of the urinary system, MIR4435-2HG is abnormally upregulated in cancer tissues and cancer cell lines of clear cell renal cell carcinoma and bladder cancer (3, 40, 41). In nervous system tumors, MIR4435-2HG is highly expressed in plasma, cancer tissues and cancer cell lines of glioma (42, 43), and in cancer tissues and cancer cell lines of glioblastoma (**Figure 3**) (44).

**Figure 4:**
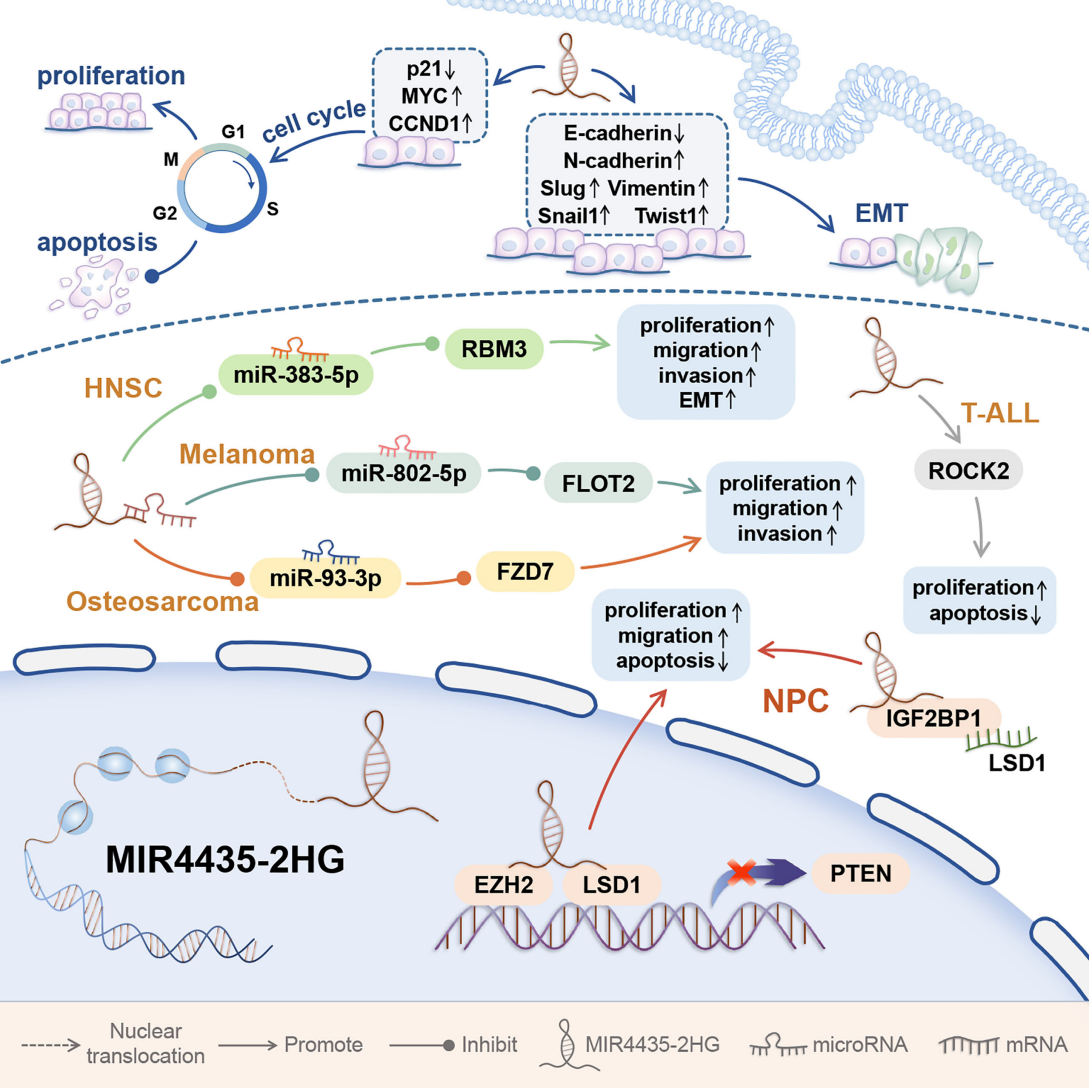
The mechanism of MIR4435-2HG affecting the behavior of tumor cells in other Systems. MIR4435-2HG can promote the progression of tumors in other systems, and affect the proliferation, invasion, migration, apoptosis, and EMT of tumor cells. Affecting EMT and cell cycle is an important mechanism for MIR4435-2HG to promote tumorigenesis. Head and neck squamous cell carcinoma (HNSC); T-cell acute lymphoblastic leukemia (T-ALL); nasopharyngeal carcinoma (NPC).

In addition, MIR4435-2HG was abnormally up-regulated in tissues and cell lines of osteosarcoma and head and neck squamous cell carcinoma (HNSC) and melanoma (45–48). MIR44435-2HG is highly expressed in childhood T-cell acute lymphoblastic leukemia (T-ALL) bone marrow and cell lines and nasopharyngeal carcinoma cell lines (**Figure 4**) (49, 50).

In the bioinformatics analysis of TCGA data, we analyzed tissue samples from prostate cancer and ALL. In experimental studies of prostate cancer, MIR4435-2HG expression was implicated in plasma and cell line samples (38, 39). In an experimental study of T-ALL, the expression of MIR4435-2HG was involved in the bone marrow and cell lines (49). Therefore, discrepancies in MIR4435-2HG results in prostate cancer and ALL may be due to sample differences. Further validation of the role of MIR4435-2HG in prostate cancer and ALL is required in the future.

The abnormal expression of MIR4435-2HG is closely related to cancer cell proliferation, apoptosis, invasion and migration. Cell cycle arrest can promote cell apoptosis and effectively inhibit cell proliferation (10). Inhibition of cell cycle progression is related to increased expression of genes that block the cell cycle and decreased expression of genes required for progression in G1, S, and M phases (2). p21 is a cyclin-dependent kinase (CDK) inhibitor, which is down-regulated in a variety of cancers. p21 can directly bind to kinases related to G1/S conversion and play a key role in cell cycle progression (21). In esophageal squamous cell carcinom (9), gastric cancer and hepatocellular carcinoma (12, 21), MIR4435-2HG knockdown can promote p21 expression. In addition, MIR4435-2HG knockdown can also reduce the expression of CCND1 and promote the cleavage of PARP and caspase-3 (12, 21). In gastric cancer and hepatocellular carcinoma (10–12, 20, 21), MIR4435-2HG knockdown can increase the proportion of G1 cells. In esophageal squamous cell carcinom (9), MIR4435-2HG knockdown can increase the ratio of G2/M-phase cells, decrease the ratio of S-phase cells, and promote cell apoptosis (**Figure 4**).

Epithelial-mesenchymal transition (EMT) usually induces the invasion and metastasis of cancer cells (24). EMT is essential in the early events of tumor cell metastasis. EMT can make cells more motile and aggressive, and it can confer cancer stem cell (CSC)-like traits on tumor cells (24). Transcription factors such as Snail1, SLUG, ZEB1, and TWIST1 can up-regulate the mesenchymal markers Vimentin and N-cadherin, and ultimately inhibit the expression of E-cadherin, a marker of epithelial status (24). In gastric cancer (10, 12), colorectal cancer (12), hepatocellular carcinoma (21), lung cancer (24), ovarian cancer (33), clear cell renal cell carcinoma (40), and HNSC (47), abnormal upregulation of MIR435-2HG can up-regulate the above-mentioned transcription factors, and ultimately promote the EMT process.

### The Relationship Between the Methylation of MIR4435-2HG CpG Sites and the Expression of MIR4435-2HG

A study has shown that lncRNA expression can be activated by DNA hypomethylation in tumors (51). In glioma, Li et al. found that the up-regulation of MIR4435-2HG may be related to its abnormal methylation through HM450K methylation microarray data (52). Here, we systematically analyzed the correlation between the expression of MIR4435-2HG and the CpG methylation of MIR4435-2HG using the Pearson method. As shown in **Figure 1C**, the methylation of cg07042346 in OV was significantly reversely correlated with the expression of MIR4435-2HG (r<-0.5, p<0.01). However, in SARC and TGCT, the CpG sites of MIR4435-2HG were significantly positively correlated with the expression of MIR4435-2HG (r>0.5, p<0.01).

## The Six Signaling Pathways Related to MIR4435-2HG in Cancers

**Figure 5:**
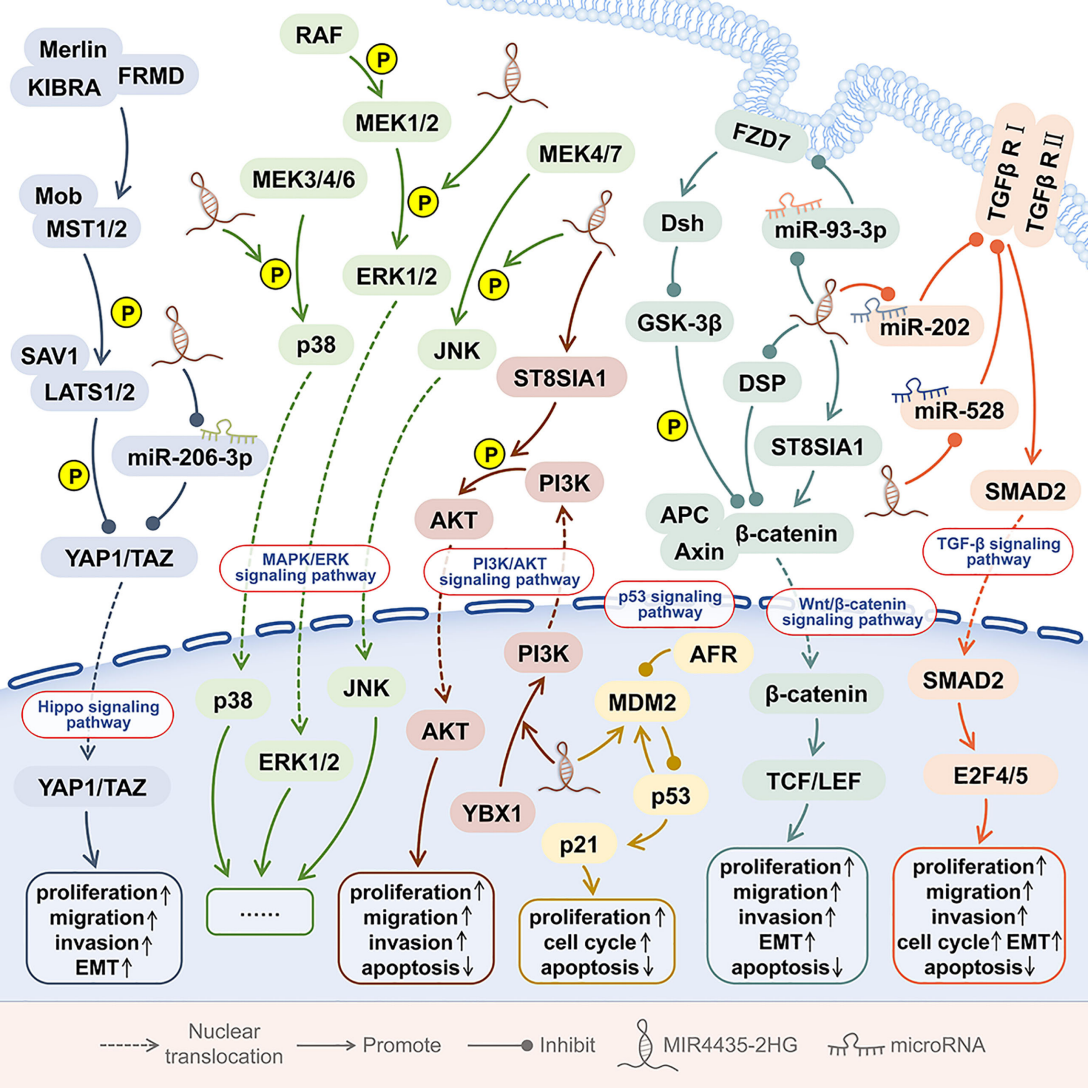
The signaling pathways involved in MIR4435-2HG. In human tumors, MIR4435 participates in at least 6 signaling pathways, including the TGF-β signaling pathway, Wnt/β-catenin signaling pathway, MDM2/p53 signaling pathway, PI3K/AKT signaling pathway, Hippo signaling pathway, and MAPK/ERK signaling pathway.

The oncogenic effect of MIR4435-2HG is related to the regulation of six signaling pathways, including the TGF-β signaling pathway, Wnt/β-catenin signaling pathway, MDM2/p53signalling pathway, PI3K/AKT signaling pathway, Hippo signaling pathway, and MAPK/ERK signaling pathway (**Figure 5**).

### The TGF-β Signaling Pathway

Transforming growth factor β (TGF-β) can bind to cell surface receptors and trigger the activation of multiple signal transduction pathways (54). In the early stage of the tumor, TGF-β can inhibit the proliferation of cancer cells, and TGF-β can promote tumor metastasis in the late stage of the tumor (55). In ovarian cancer, TGF-β targeted therapy needs to be carried out cautiously according to the cancer stage (32). The activation of the TGF-β signaling pathway is widely present in the development of tumors (17).

Overexpression of MIR4435-2HG can up-regulate TGF-β1 and promote the metastasis of 7 kinds of tumors, including oral squamous cell carcinoma (OSCC) (7), gastric cancer (12), colorectal adenocarcinoma (17), and non-small cell lung cancer (27, 28, 30), ovarian cancer (32), prostate cancer and glioma (38, 43). In addition, in OSCC and colorectal adenocarcinoma (7, 17), the up-regulation of MIR4435-2HG and TGF-β1 expression can promote tumor growth and metastasis. TGF-β is the main regulator of EMT and a key marker for the metastasis and progression of different malignant tumors (56). In gastric cancer and non-small cell lung cancer (12, 27), the upregulation of MIR4435-2HG and TGF-β1 can promote the EMT of tumor cells. In non-small cell lung cancer, the up-regulation of MIR4435-2HG and TGF-β1 is also closely related to postoperative tumor recurrence (28).

In OSCC (7), non-small cell lung cancer (27, 28, 30), ovarian cancer (32), prostate cancer (38), and glioma (43), the expression levels of MIR4435-2HG and TGF-β1was positively correlated with each other, while no correlation between MIR4435-2HG and TGF-β1 was found in plasma of healthy persons. Overexpression of MIR4435-2HG can up-regulate the expression of TGF-β1, while exogenous TGF-β1 treatment has no effect on the expression of MIR4435-2HG. In gastric cancer (10), colorectal cancer (15), lung cancer (24, 29), ovarian cancer (31), breast cancer (35), and osteosarcoma (45), the overexpression of MIR4435-2HG can increase β-catenin and promote tumorigenesis. β-catenin has been shown to interact with TGF-β (57). Therefore, β-catenin may mediate the interaction between MIR4435-2HG and TGF-β1 (32, 38, 43), but this still needs further research and verification.

### The Wnt/β-Catenin Signaling Pathway

β-catenin is located in the cell nucleus, and by controlling gene transcription, it can promote canceration and cancer cell metastasis, and induce cancer cell stemness and drug resistance (15, 24). β-catenin is a well-known oncogene and plays a key role in regulating the Wnt signaling pathway. The Wnt/β-catenin signaling pathway plays a key role in the growth of a variety of tumors (58). The increased cytoplasmic β-catenin content is a sign of the abnormal activation of Wnt/β-catenin pathway. β-catenin plays a key role in regulating the Wnt signaling pathway, and it controls the transcription of target genes in the nucleus (15).

In stomach cancer (10), colorectal cancer (15), lung cancer (24, 29), ovarian cancer (31), breast cancer and osteosarcoma (35, 37, 45), MIR4435-2HG can up-regulate the expression of β-catenin proportionally. In gastric cancer, desmoplakin (DSP) is the most abundant desmosomal protein. MIR4435-2HG can bind to DSP and inhibit DSP and its cascade reaction, thereby activating Wnt/β-catenin signal transduction, promoting tumor growth, metastasis, and EMT (10).

In lung cancer, MIR4435-2HG up-regulates β-catenin, promotes tumor growth, metastasis and EMT both *in vivo* and *in vitro*, and maintains the stemness of cancer cells (24). In non-small cell lung cancer, MIR4435-2HG can up-regulate β-catenin to promote cell proliferation and inhibit cell apoptosis (29). In ovarian cancer, overexpression of MIR4435-2HG significantly promotes the expression of β-catenin and promotes the growth, invasion, and migration of tumor cells (31). In addition, in breast cancer, MIR4435-2HG promotes tumor growth, invasion, metastasis, and EMT by activating Wnt/β-catenin signal transduction, and inhibits cell apoptosis (35); meanwhile, MIR44352HG knockdown can decreases the expression of total and nuclear β-catenin, reduces the expression of anti-apoptotic marker (Bcl2), proliferation marker (PCNA), and mesenchymal markers (N-cadherin, vimentin, and ZEB1), upregulates the cleaved PARP and the epithelial marker (E-cadherin), and activate caspase 3 and Bax of the apoptotic pathway (35).

Frizzled family receptor 7 (FZD7) is a Wnt signaling receptor, which is involved in the maintenance of cancer cell stemness and cancer progression. In triple-negative breast cancer (TNBC), overexpression of MIR4435-2HG promotes frizzled homolog 7 (FZD7) expression in cells, thereby activating the Wnt/β-catenin signaling pathway (37). In osteosarcoma, through the miR-93-3p/FZD7 axis, MIR4435-2HG can also up-regulate FZD7 and activate the Wnt/β-catenin signaling pathway, thereby promoting the proliferation, invasion, and migration of osteosarcoma cells (45). In addition, in prostate cancer tissues and cells, MIR4435-2GH increases the expression levels of β-catenin, p-FAK, p-AKT, c-MYC, and CCND1 by up-regulating ST8SIA1 (39).

In the above tumors, MIR4435-2HG can promote the expression of β-catenin, but the activation of Wnt/β-catenin signal transduction has no effect on the expression of MIR4435-2HG. Therefore, MIR4435-2HG is an upstream activator of the Wnt/β-catenin signaling pathway, which plays a role in the occurrence and development of cancer.

### The MDM2/p53 Signaling Pathway

MDM2/p53 is one of the important signaling pathways, which can regulate cell growth and cell cycle (59). Mouse double minute 2 (MDM2) is an E3 ubiquitin ligase, which not only inhibits the transcriptional activity of p53, but also promotes the ubiquitination and degradation of p53 (9). p53 is an important tumor suppressor, which is activated in response to various stresses, thereby promoting cell apoptosis (9).

In esophageal squamous cell carcinoma tissues, both MIR4435-2HG and MDM2 were significantly up-regulated. Knockdown of MIR4435-2HG resulted in G2/M phase arrest in esophageal squamous cell carcinom cell lines (Eca-109 and TE-1), as well as decreased expression of MDM2, and increased expression of downstream p53 and p21 (9). The above shows that MIR4435-2HG promotes cell proliferation and cell cycle and inhibits cell apoptosis by regulating the MDM2/p53 signaling pathway.

### The PI3K/AKT Signaling Pathway

When PI3K binds to growth factor receptors such as EGFR, RAS and PTEN, it can change the protein structure of AKT and activate AKT and its downstream effectors, thereby regulating cell proliferation, differentiation, apoptosis, and migration (60). The PI3K/AKT pathway is involved in the occurrence and development of a variety of cancers (60).

In the nucleus of hepatocellular carcinoma (HCC) cells, the interaction between MIR4435-2HG and the DNA binding protein Y-box binding protein 1 (YBX1) can enhance the binding of YBX1 to the PI3K promoter, thereby promoting the transcription of PI3K. MIR4435-2HG can activate the PI3K/AKT pathway, promote the proliferation and migration of HCC cells, and promote tumor growth and metastasis in mice. In the cytoplasm of HCC, MIR4435-2HG can interact with YBX1, up-regulate Snail1, and promote tumor progression (22). In prostate cancer tissues and cell lines, MIR4435-2HG promotes ST8SIA1 and up-regulates p-AKT levels (39).

In osteosarcoma cell lines (MG-63 and U2OS), MIR4435-2HG can up-regulate the protein levels of p-PI3K and p-AKT, suggesting that MIR4435-2HG may activate the PI3K/AKT pathway to promote the growth of osteosarcoma cells, Invasion, migration and apoptosis (46).

### The Hippo Signaling Pathway

YAP1 is a Hippo signaling pathway gene, which is amplified in a variety of human cancers (61). YAP1 is a transcriptional regulator that is widely activated in human malignancies and can induce the proliferation, metastasis, stemness and chemotherapy resistance of cancer cells (61). In colorectal cancer, MIR4435-2HG binds miR-206-3p to up-regulate downstream YAP1 expression. MIR4435-2HG promotes the proliferation, invasion, migration and EMT of colorectal cancer cells by activating the Hippo signaling pathway, and promotes tumor growth *in vivo* (1).

### The MAPK/ERK Signaling Pathway

The MAPK/extracellular signal-regulated kinase (ERK) signaling pathway is highly conserved. The MAPK/ERK signaling pathway involves a variety of biological events, including metabolic reprogramming, cell proliferation, survival, and differentiation (62). In the MAPK/ERK signaling pathway, mutations and dysfunctions of key genes are very common events in various human malignancies (62). In HCC cells, MIR4435-2HG can promote the phosphorylation of ERK, p38, and c-Jun N-terminal kinase (JNK), and activate the MAPK/ERK signaling pathway, thereby promoting HCC cell proliferation, cell cycle progression, and survival (20).

## The ceRNA Network of MIR44350-2HG

The competing endogenous RNA (ceRNA) hypothesis describes that lncRNA and mRNA may bind to the same miRNA through their miRNA response element (MRE) (52). lncRNAs and miRNAs are the two main subgroups of ncRNAs, and both have been shown to be key players in cancer biology (19).

**Figure 6:**
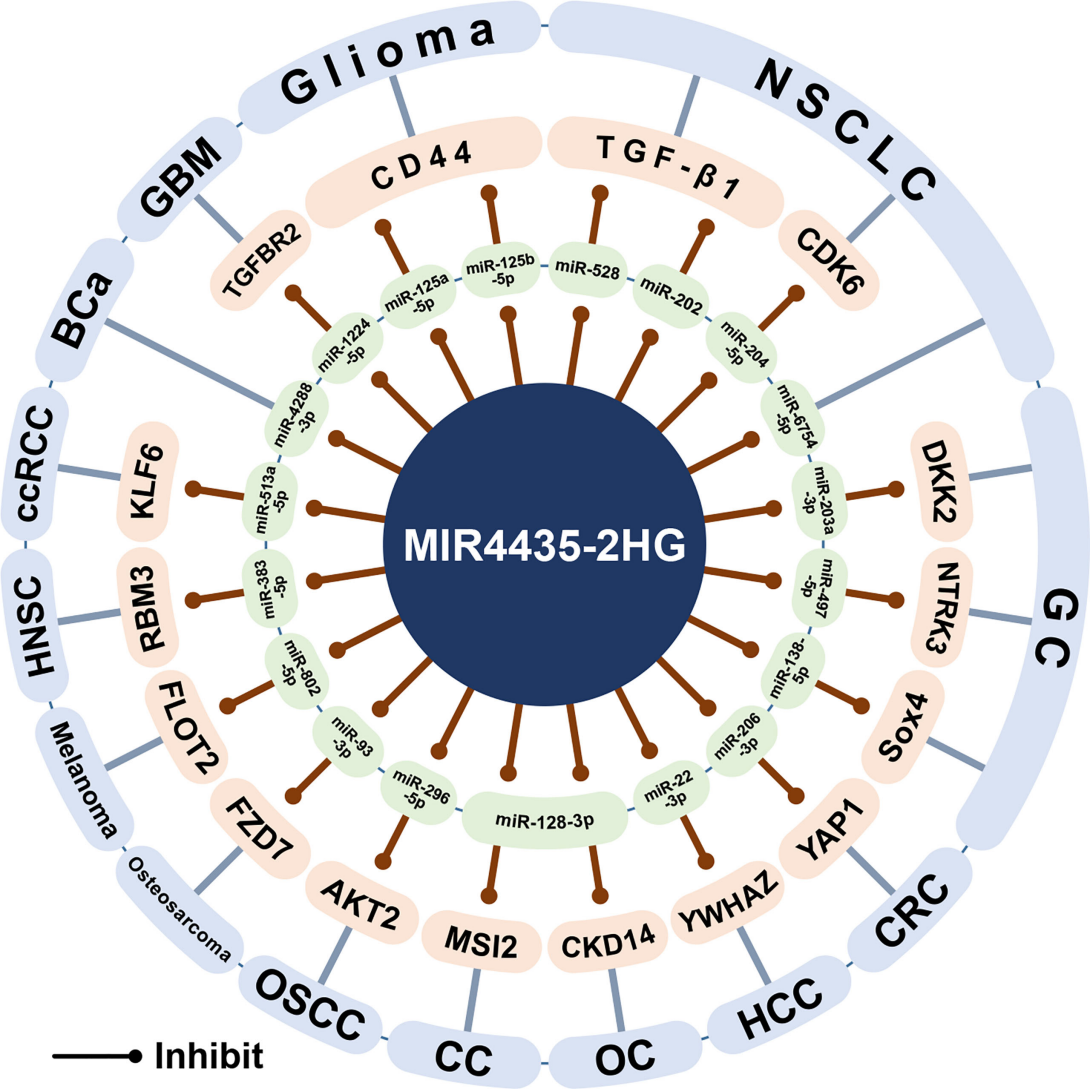
The ceRNA network of MIR4435-2HG. MIR4435-2HG can interact with 19 miRNAs in at least 14 cancers and osteoarthritis, and regulate the expression of its downstream target genes. HCC, Hepatocellular carcinoma; CRC, Colorectal cancer; OSCC, Oral squamous cell carcinoma; GC, Gastric cancer; NSCLC, Non-small cell lung cancer; GBM, Glioblastoma; BCa, Bladder cancer; ccRCC, clear cell renal cell carcinoma; OC, Ovarian cancer; CC, Cervical cancer; HNSC, Head and neck squamous cell carcinoma.

Here, this article outlines the ceRNA network centered on MIR4435-2HG and its biological significance (**Figure 6**). MIR4435-2HG can be used as the ceRNA of 20 miRNAs, 19 of which are found in 14 cancers, including miR-22-3p (18), miR-206-3 (1), miR-296-5p (1), miR-497-5p (11), miR-138-5p (53), miR-203a-3p (13), miR-6754-5p (25), miR-204-5p (26), miR-528 (30), miR-202 (30), miR-125a-5p (52), miR125b-5p (52), miR-1224-5p (44), miR-4288-3p (41), miR-513a-5p (3), miR-128- 3p (33), miR-93-3p (45), miR-802-5p (48), and miR-383-5p (47). In addition, in osteoarthritis, MIR4435-2HG was found to sponge miR-510-3p (63).

In the digestive system, MIR4435-2HG can up-regulate YWHAZ and YAP1 by competitively binding miR-22-3p and miR-206-3p to promote the progression of hepatocellular carcinoma and colorectal cancer, respectively (1, 18). In oral squamous cell carcinoma, the MIR4435-2HG/miR-296-5p axis inhibits AKT2, thereby promoting the expression of Snail1, an important transcription factor regulating EMT (64). In gastric cancer, MIR4435-2HG has been shown to promote gastric cancer progression through the miR-497-5p/NTRK3 axis (11) and the miR-138-5p/Sox4 axis (53). However, another study showed that the expression of MIR4435-2HG decreased in gastric cancer, and DKK2 was down-regulated through the MIR4435-2HG/miR-203a-3p axis to inhibit tumor progression (13). It is worth noting that the results of three gastric cancer studies have shown that the expression of MIR4435-2HG in gastric cancer cell lines (HGC-27, BGC-823, SGC-7901, SNU5, AGS, and MGC-803) is higher than that in gastric mucosal cell lines (GES-1) (10–12). However, one study showed that the expression of MIR4435-2HG in four gastric cancer cell lines (HGC-27, BGC-823, SGC-7901, and MKN-45) was lower than that of gastric mucosal cell line (GES-1) (13).

In non-small cell lung cancer, MIR4435-2HG can sponge miR-528 and miR-202, and subsequently up-regulate TGF-β1 to promote tumor growth (30). In addition, MIR4435-2HG/miR-204-5p/CDK6 axis and MIR4435-2HG/miR-6754-5p axis can promote the growth and invasion of non-small cell lung cancer (25, 26). In gliomas, MIR4435-2HG/miR-125a-5p axis and MIR4435-2HG/miR125b-5p axis can up-regulate CD44 and promote tumor progression (52). In glioblastoma, MIR4435-2HG competitively binds miR-1224-5p, thereby up-regulating TGFBR2 and promoting tumor growth (44). In bladder cancer, MIR4435-2HG can sponge miR-4288-3p and promote tumor growth and invasion (41). In clear cell renal cell carcinoma, MIR4435-2HG/miR-513a-5p promotes tumorigenesis and development by promoting the expression of KLF6 (3). In ovarian cancer and cervical cancer (33, 34), MIR4435-2HG can competitively bind miR-128-3p and up-regulate CKD14 and MSI2, thereby promoting tumor progression. In osteosarcoma, the MIR4435-2HG/miR-93-3p axis can up-regulate FZD7 and promote tumor progression (45). In melanoma, the MIR4435-2HG/miR-802-5p axis can promote FLOT2 expression and promote tumor growth and invasion (48). In HNSC, the MIR4435-2HG/miR-383-5p axis can up-regulate RBM3, thereby promoting tumor progression (47). In addition, in osteoarthritis, low expression of MIR4435-2HG can attenuate the MIR4435-2HG/miR-510-3p/IL-17A axis signal and activate the NF-κB signaling pathway, thereby mediating the process of osteoarthritis (63).

In summary, the carcinogenic effect of MIR4435-2HG is through sponging miRNAs to regulate the expression of the downstream target genes. In addition, in osteoarthritis, the low expression of MIR4435-2HG promotes the process of osteoarthritis through the ceRNA network.

## The Relationship Between MIR4435-2HG and Clinicopathological Characteristics

As shown in **Table 2**, the abnormal up-regulation of MIR4435-2HG is closely related to the clinicopathological characteristics of 11 tumors. In tumors of the digestive system, high expression of MIR4435-2HG is associated with larger tumors, advanced TNM staging and lymph node metastasis in esophageal squamous cell carcinom (9), gastric cancer (10–12), colorectal cancer (1, 14, 16), and hepatocellular carcinoma (18–20, 23). In esophageal squamous cell carcinom, the upregulation of MIR4435-2HG is also related to tumor differentiation and advanced UICC (Union for International Cancer Control) staging (8, 9). In colorectal cancer, the up-regulated MIR4435-2HG is positively correlated with tumor grade and patient age (15). In hepatocellular carcinoma, high expression of MIR4435-2HG is significantly correlated with distant metastasis, advanced Edmondson grade, incomplete encapsulation, microvascular invasion, and advanced BCLC stage (18, 20, 22).

**Table 2:** Clinicopathologic significance of MIR4435-2HG in human cancers.

System	Tumor type	Sample size	Expression	Clinicopathological features	Prognostic value	Diagnostic value	Ref.
Digestive system	ESCC	50 patients	Upregulation	Positively associated with tumor diferentiation, large tumor size, advanced TNM stage, and lymph node metastasis	Negatively associated with OS		(9)
		175 patients	Upregulation	Positively associated with advanced UICC stage and lymph node metastasis	Negatively associated with DFS and OS		(8)
	GC	57 patients	Upregulation	Positively associated with advanced TNM stage			(10)
		150 patients	Upregulation	Positively associated with advanced TNM stage	Negatively associated with OS		(11)
		72 patients	Upregulation	Positively associated with large tumor size, advanced TNM stage, and lymph node metastasis		AUC = 0.746, sensitivity = 0.53, specificity = 0.92 (in tissue); AUC = 0.831, sensitivity = 0.80, specificity = 0.70 (in serum)	(12)
		60 patients	Upregulation	Positively associated with advanced TNM stage and lymph node metastasis	Negatively associated with OS		(53)
	CRC	90 patients	Upregulation	Positively associated with large tumor size and advanced TNM stage	Negatively associated with DFS and OS		(1)
		102 patients	Upregulation	Positively associated with large tumor size, advanced TNM stage, and lymph node metastasis	Negatively associated with DFS and OS		(14)
		70 patients	Upregulation	Positively associated with tumor grade and age		AUC = 0.81, specificity = 0.8095, sensitivity = 0.7273	(15)
	COAD	86 patients	Upregulation		Negatively associated with OS	AUC: = 0.9065 (in serum)	(17)
	Colon cancer	46 patients	Upregulation	Positively associated with large tumor size		AUC = 0.8481 (in serum, I-II stage)	(16)
	HCC	64 patients	Upregulation	Positively associated with large tumor size			(19)
		22 patients	Upregulation	Positively associated with advanced TNM stage and distant metastasis	Negatively associated with OS		(18)
		49 patients	Upregulation	Positively associated with large tumor size, advanced Edmondson grade, advanced TNM stage, and lymph node metastasis	Negatively associated with OS		(20)
		88 patients	Upregulation	Positively associated with encapsulation incomplete, microvascular invasion, advanced BCLC stage, and advanced TNM stage	Negatively associated with RFS and OS		(22)
		73 patients	Upregulation	Positively associated with large tumor size and advanced TNM stage	Negatively associated with OS		(23)
		58 patients	Upregulation			AUC = 0.910, sensitivity = 0.76, specificity = 0.96 (in serum)	(21)
Reproductive system	BC	195 patients	Upregulation	Negatively associated with HR status	Negatively associated with DFS		(36)
	TNBC	68 patients	Upregulation	Positively associated with large tumor size and advance TNM stage	Negatively associated with OS	AUC = 0.8927 (in plasma)	(37)
		72 patients	Upregulation			AUC = 0.7980 (in plasma, I-II stage)	(6)
	OC	42 patients	Upregulation	Positively associated with large tumor size, advanced FIGO stage and lymph node metastasis	Negatively associated with OS		(33)
		58 patients	Upregulation	Positively associated with large tumor size and distant metastasis	Negatively associated with OS	AUC = 0.9082 (in serum)	(31)
		28 patients	Upregulation	Positively associated with distant metastasis		AUC = 0.8824 (in plasma, I-II stage)	(32)
	CC	59 patients	Upregulation	Positively associated with advanced FIGO stage and lymph node metastasis			(34)
	PCa	68 patients	Upregulation		Negatively associated with OS		(38)
Respiratory system	LC	52 patients	Upregulation	Positively associated with advanced TNM stage and lymph node metastasis	Negatively associated with OS		(24)
	NSCLC	39 patients	Upregulation	Positively associated with advanced TNM stage and lymph node metastasis			(25)
		138 patients	Upregulation	Positively associated with distant metastasis			(27)
		128 patients	Upregulation		Positively associated with distant recurrence		(28)
		Number not shown	Upregulation		Negatively associated with OS		(26)
		88 patients	Upregulation	Positively associated with large tumor size and smoking habit	Negatively associated with OS	AUC = 0.8686 (in tissue); AUC = 0.8569 (in serum)	(29)
Nervous system	Glioma	34 metastatic glioma patients and 32 non-metastatic glioma patients	Upregulation			AUC = 0.8640 (metastatic glioma) (in plasma)	(42)
	GBM	40 patients	Upregulation		Negatively associated with OS		(44)
Urinary system	ccRCC	118 patients	Upregulation	Positively associated with large tumor size, advanced Fuhrman grade, and advanced TNM stage	Negatively associated with RFS and OS	AUC = 0.946 (in tissue)	(40)
Others	T-ALL	32 patients	Upregulation			AUC = 0.8954 (in bone marrow)	(49)
	HNSC	18 patients	Upregulation	Positively associated with advanced TNM stage	Negatively associated with DFS and OS		(47)
	Osteosarcoma	36 patients	Upregulation	Positively associated with large tumor size, advance TNM stage and distant metastasis	Negatively associated with RFS and OS		(45)

ESCC, esophageal squamous-cell carcinoma; GC, gastric cancer; CRC, colorectal cancer; HCC, hepatocellular carcinoma; BC, breast cancer; TNBC, triple-negative breast cancer; OC, ovarian cancer; CC, cervical cancer; PCa, prostate carcinoma; LC, lung cancer; NSCLC, nonsmall cell lung cancer; GBM, glioblastoma; ccRCC, clear cell renal cell carcinoma; T-ALL, T-cell acute lymphoblastic leukemia; HNSC, head and neck squamous cell carcinoma; TNM, tumor-node-metastasis; UICC, Union for International Cancer Control; BCLC, Barcelona Clinic Liver Cancer; FIGO, Federation of Gynecology and Obstetrics; HR, hormone receptor; DFS, isease-free survival; OS, overall survival; RFS, recurrence-free survival; AUC, area under the curve.

In breast cancer, MIR4435-2HG is negatively correlated with hormone receptor levels (36). In TNBC, the up-regulated MIR4435-2HG also points to larger tumors and higher TNM stages (37). In ovarian cancer and cervical cancer (33, 34), the high expression level of MIR4435-2HG is also related to advanced FIGO (Federation of Gynecology and Obstetrics) stage and lymph node metastasis. In ovarian cancer, MIR4435-2HG is also closely related to larger tumors and distant metastasis of tumors (31, 33). In lung cancer, high expression of MIR4435-2HG is significantly associated with larger tumors, higher TNM stages, stronger lymph node metastasis, and distant metastasis of the tumor (24, 25, 27, 29). The expression level of MIR4435-2HG in lung cancer tissues and serum of lung cancer patients is significantly positively correlated with tumor size and smoking habits (29). In addition, in clear cell renal cell carcinoma (40), HNSC (47), and osteosarcoma (45), MIR4435-2HG was found to be closely related to advanced TNM stages. In addition, MIR4435-2HG also points to large tumor size and advanced Fuhrman grade in clear cell renal cell carcinoma and distant metastasis of tumors in osteosarcoma (40, 45).

## The Prognostic and Diagnostic Value of MIR4435-2HG

Abnormal up-regulation of MIR4435-2HG has potential value for cancer diagnosis and prognosis. The high expression of MIR4435-2HG is associated with a significant reduction in the overall survival (OS) of patients with 12 types of tumors (**Table 2**), including esophageal squamous cell carcinoma (8, 9), gastric cancer (11), colorectal cancer (1, 14, 17), hepatocellular carcinoma (18, 20, 22, 23), triple-negative breast cancer (TNBC) (37), ovarian cancer (31, 33), prostate cancer (38), lung cancer (24, 26, 29), glioblastoma (44), clear cell renal cell carcinoma (40), HNSC (47), and osteosarcoma (45). Among them, high MIR4435-2HG expression is significantly associated with shorter disease-free survival (DFS) in patients with esophageal squamous cell carcinoma (8), colorectal cancer (1, 14), breast cancer (36), or HNSC (47). In addition, high expression of MIR4435-2HG is also associated with shorter recurrence-free survival (RFS) in patients with hepatocellular carcinoma (22), clear cell renal cell carcinoma (40), or osteosarcoma (45). MIR4435-2HG is also positively correlated with postoperative distant recurrence in patients with non-small cell lung cancer (28).

As shown in **Table 2**, the high expression level of MIR4435-2HG in the tumor tissues and/or blood (whole blood, serum, and plasma) of cancer patients has proved to be of great diagnostic value in 9 cancers. In the tissues and serum of gastric cancer (12), colorectal cancer (15, 17), and non-small cell lung cancer (29), high expression of MIR4435-2HG can distinguish tumor patients from normal controls. In hepatocellular carcinoma serum (21), clear cell renal cell carcinoma tissue (40), and childhood T-ALL bone marrow (49), higher expression levels of MIR4435-2HG can distinguish tumor patients from normal controls. It is worth noting that the high expression of MIR4435-2HG in the serum of colon cancer and the plasma of TNBC and ovarian cancer can effectively distinguish patients with early-stage tumors (stage I-II) and healthy controls Group (6, 16, 32), suggesting that MIR4435-2HG may be used as an early diagnostic marker for these three tumors. In addition, in gliomas, the highly expressed MIR4435-2HG can distinguish metastatic tumors from healthy controls, but cannot effectively distinguish non-metastatic gliomas from normal healthy controls. This indicates that MIR4435-2HG may be involved in the process of glioma metastasis and can be used to diagnose glioma metastasis (42).

## MIR4435-2HG and Tumor Drug Treatment

**Figure 7:**
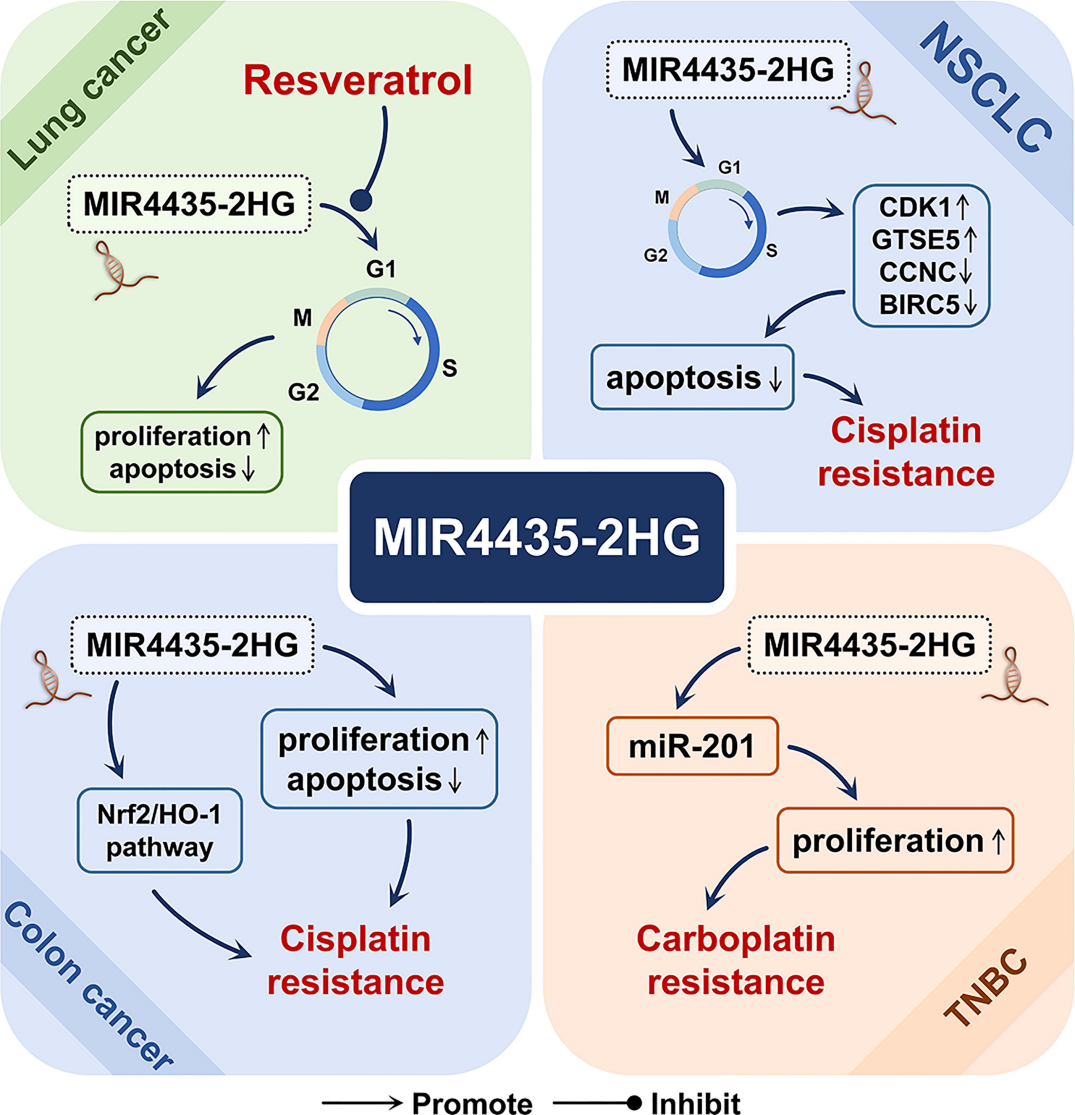
The role of MIR4435-2HG in cancer drugs. In lung cancer, MIR4435-2HG may be involved in the inhibitory effect of resveratrol on the growth of lung cancer cells. In non-small cell lung cancer (NSCLC) and colon cancer, MIR4435-2HG may be a driving factor for cisplatin resistance. In triple-negative breast cancer (TNBC), MIR4435-2HG may be involved in the development of carboplatin resistance.

MIR4435-2HG has also been shown to be involved in the mechanism of action of a variety of tumor treatment drugs, including resveratrol for the treatment of lung cancer (2), cisplatin for non-small cell lung cancer and colon cancer (4, 5), and carboplatin for three-negative breast cancer (**Figure 7**) (6).

Resveratrol is a natural polyphenol, found in various plants and Chinese herbal medicines. Due to its relatively low toxicity, it can promote cancer by targeting a variety of signaling molecules for cell survival and tumor growth. It is considered an ideal chemopreventive agent (2). As an oncogenic lncRNA, MIR4435-2HG is highly expressed in lung cancer cell lines, and its expression is down-regulated after resveratrol treatment, thereby inhibiting the proliferation and growth of lung cancer cells (2).

Cisplatin is widely used in the treatment of various cancers, but the emergence of cisplatin resistance is a serious clinical problem (5). In non-small cell lung cancer, MIR4435-2HG knockdown can reduce the cisplatin resistance and cell viability of the cisplatin-resistant cell line A549/DDP, and cause cell cycle arrest, which significantly increases the ratio in the G0/G1 phase. Meanwhile, MIR4435-2HG knockdown positively induced the expression of apoptosis-related factors (CCNC and BIRC5), and inhibited the expression of cell cycle-related factors (CDK1 and GTSE5), thereby promoting cell apoptosis (4). In colon cancer, MIR4435-2HG is highly expressed in the cisplatin-resistant cell line HCT116R, and MIR4435-2HG knockdown can significantly restore the sensitivity of cells to cisplatin, inhibit cell proliferation, and promote cell apoptosis (5). In addition, in colon cancer, MIR4435-2HG knockdown can reduce the transcription levels of key molecules (Nrf2 and HO-1) in the oxidative stress pathway (5).

Carboplatin is a cisplatin derivative with broad-spectrum anti-tumor activity. It can be used as a single drug or combined to treat multiple tumors (65). In triple-negative breast cancer (TNBC), overexpression of MIR4435-2HG and miR-21 can promote the proliferation of cancer cells treated with carboplatin, improve the viability of cancer cells, and induce chemotherapy resistance (6).

In summary, MIR4435-2HG may be involved in the inhibitory effect of resveratrol on the growth of lung cancer cells and may be an important driving factor for cisplatin and carboplatin resistance.

## Conclusions and Perspectives

MIR4435-2HG is a lncRNA with great potential, which can be used as a diagnostic and prognostic biomarker for a variety of tumors, and a therapeutic target for a variety of tumors. MIR4435-2HG was abnormally up-regulated in tumor tissues and cell lines as an oncogene in 18 tumors, and its overexpression was also detected in the blood, plasma, or serum of 9 tumors. At the same time, MIR4435-2HG is closely related to the clinical characteristics and poor prognosis of 12 tumors. This may mean that MIR4435-2HG can be highly expressed and detected in human blood besides tumor tissues. In the future, the relationship between MIR4435-2HG and tumor development can be studied in more tumors. In addition, there is only one study on the methylation of MIR4435-2HG in gliomas (52), and the mechanism of MIR4435-2HG overexpression in these tumors has not been elucidated. Epigenetic research can provide hints for elucidating the molecular mechanism of MIR4435-2HG.

MIR4435-2HG can participate in at least 6 signal pathways and form a ceRNA network with miRNAs to promote the occurrence and development of tumors. In tumors, MIR4435-2HG can participate in the regulation of signaling pathways and affect different biological processes of tumors. For example, non-small cell lung cancer is involved in TGF-β signaling (27, 28, 30) and Wnt/β-catenin signaling (29). Colorectal cancer is involved in the Wnt/β-catenin signaling pathway (15) and the Hippo signaling pathway (1). This may provide ideas for exploring new tumor treatment strategies. However, the specific mechanism of MIR4435-2HG in the pathway has not been well explained. Meanwhile, the existing research on MIR4435-2HG mostly focuses on the “lncRNA-miRNA-mRNA” axis, however, the research on the relationship between other non-coding RNAs of MIR4435-2HG is still lacking. For example, MIR4435-2HG and BCL2L11 genes co-localize to chr2 q13, and the lncRNA Morrbid (a myeloid RNA regulator of BCL2L11-induced cell death) is involved in the regulation of N-ras splicing in mouse hepatocytes and is associated with tumorigenesis (66). In the future, it is necessary to further study the regulatory mechanism of MIR4435-2HG and improve its ceRNA network. In addition, the expression of MIR4435-2HG may also be closely associated with nearby genetic variants. For example, the rs17041869 site located in the enhancer of BCL2L11 can regulate the expression of the BCL2L11 gene near MIR4435-2HG (67), suggesting the need to explore genetic variants associated with MIR4435-2HG in the future.

In addition, it needs to be further explored for the application of MIR4435-2HG in the blood of tumor patients in the diagnosis and prognosis of tumors. The connection between MIR4435-2HG and tumor treatment drugs lays the foundation for the clinical treatment of tumors. In the future, it is necessary to test the role of MIR4435-2HG in the treatment of more cancer drugs.

## Author Contributions

SD, CY, and CZ contributed to the conception, design and final approval of the submitted version. CZ and ZX collected and analyzed literature. CZ, ZX, L-hZ, CY, and SD contributed to manuscript writing. All the authors conceived and gave the approval of the final manuscript.

## Funding

The research was supported by National Natural Science Foundation of China (32100521) and Qiantang Scholar Fund in Zhejiang University City College.

## Conflict of Interest

The authors declare that the research was conducted in the absence of any commercial or financial relationships that could be construed as a potential conflict of interest.

## Publisher’s Note

All claims expressed in this article are solely those of the authors and do not necessarily represent those of their affiliated organizations, or those of the publisher, the editors and the reviewers. Any product that may be evaluated in this article, or claim that may be made by its manufacturer, is not guaranteed or endorsed by the publisher.
